# Maintenance of a Living Understory Enhances Soil Carbon Sequestration in Subtropical Orchards

**DOI:** 10.1371/journal.pone.0076950

**Published:** 2013-10-08

**Authors:** Zhanfeng Liu, Yongbiao Lin, Hongfang Lu, Mingmao Ding, Yaowen Tan, Shejin Xu, Shenglei Fu

**Affiliations:** 1 Key Laboratory of Vegetation Restoration and Management of Degraded Ecosystems, South China Botanical Garden, Chinese Academy of Sciences, Guangzhou, China; 2 Guangzhou Fruit Research Institute, Guangzhou, China; DOE Pacific Northwest National Laboratory, United States of America

## Abstract

Orchard understory represents an important component of the orchards, performing numerous functions related to soil quality, water relations and microclimate, but little attention has been paid on its effect on soil C sequestration. In the face of global climate change, fruit producers also require techniques that increase carbon (C) sequestration in a cost-effective manner. Here we present a case study to compare the effects of understory management (sod culture vs. clean tillage) on soil C sequestration in four subtropical orchards. The results of a 10-year study indicated that the maintenance of sod significantly enhanced the soil C stock in the top 1 m of orchard soils. Relative to clean tillage, sod culture increased annual soil C sequestration by 2.85 t C ha^-1^, suggesting that understory management based on sod culture offers promising potential for soil carbon sequestration. Considering that China has the largest area of orchards in the world and that few of these orchards currently have sod understories, the establishment and maintenance of sod in orchards can help China increase C sequestration and greatly contribute to achieving CO_2_ reduction targets at a regional scale and potentially at a national scale.

## Introduction

Substantial research and practical experience has demonstrated that understory vegetation plays an important role in driving forest ecosystem processes and functioning. As an ecological filter, understory vegetation can affect both aboveground processes (such as tree seedling regeneration, forest succession, species diversity and stand productivity) and belowground processes (such as decomposition, soil nutrient cycling and soil water conservation) [1,2]. Removal experiments in forest ecosystems have also revealed that the loss of species and functional group in understory vegetation often reduces key ecosystem processes including soil nutrient cycling, microbial community activity and composition, and litter decomposition [3-6].

Agroforestry systems play a central role in the global carbon (C) cycle and contain approximately 12% of the Earth’s terrestrial C [7]. The C sequestration potential of agroforestry systems has attracted worldwide attention following the recognition by the Kyoto Protocol that agroforestry can help reduce greenhouse gases [8,9]. Agroforestry is a management system that integrates trees with farms in an agricultural landscape and usually encompasses a wide variety of understory vegetation management methods. Many studies have shown that management of understory vegetation can greatly influence soil fertility, tree nutrition, and fruit quality [10]. Because C sequestration in agroforestry systems is largely dependent on how the system is managed [8], understory vegetation management is expected to influence soil C sequestration by affecting soil nutrient cycling and system productivity [11]. Most of the available reports on C sequestration in agroforestry system are estimates of C stocks [9], but little has been reported regarding how understory vegetation management affects soil C sequestration in agroforestry systems, especially in subtropical or tropical orchards.

**Figure 1:**
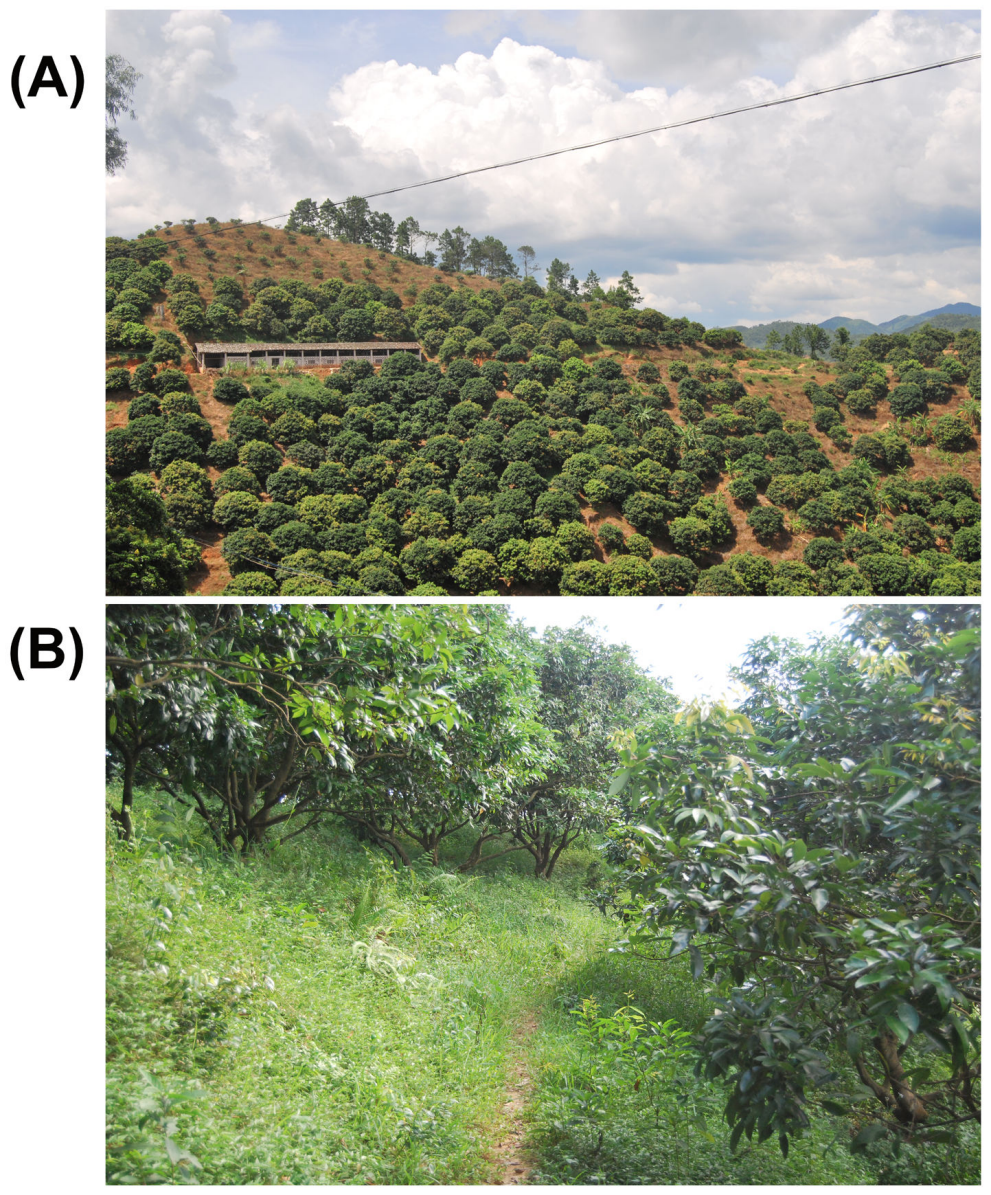
Understory vegetation management in orchard systems in Guangdong Province, southern China: (A) Sod culture and (B) Clean tillage. The image used in the figure is not the original image used in the study and only used for illustrative purposes.

Orchard understories perform a number of functions related to soil quality, water relations, and microclimate, and these functions are greatly affected by management [12]. As a traditional management practice in orchard ecosystem, clean tillage usually includes the frequent control of understory weeds to reduce the competition for water and nutrients between fruit trees and weeds (Figure 1A). However, frequent tillage can destroy soil structure and cause substantial soil erosion and nutrient loss. With increased attention on sustainable orchard management and organic fruit production, sod culture has been widely accepted throughout the world (Figure 1B); sod culture can reduce soil erosion, improve soil fertility and the orchard microclimate, and increase fruit production and quality [13]. Soil carbon levels can be considerably increased if management options favors increases in carbon inputs (i.e., increases in primary production and litter deposition) and reductions in carbon losses (i.e., reductions in decomposition, leaching, and erosion [14]). A growing body of evidence has also revealed that sod culture in orchards can greatly increase soil organic matter content, soil nutrient availability, and soil biological activity; can improve the microclimate; and can reduce soil erosion [15-17]. Although researchers have noted that sod culture has the potential to increase soil C sequestration in orchards [18], our understanding of how sod culture affects the intensity and rate of soil carbon sequestration in orchard ecosystems is limited.

As a major type of agroforestry system, orchards have been rapidly developed in China since the 1990s. By 2009, the area occupied by orchard in China had increased to 11.14 million hectares, and annual fruit production had increased to 200 million tons, making China the leading countries in orchard production [19]. Here, we compare the effects of maintaining a living understory, i.e., sod culture, vs. clean tillage on soil C sequestration and soil fertility in subtropical orchards in South China. Finally, we discuss the feasibility and implication of understory management as an option for CO_2_ mitigation in subtropical orchard systems.

## Materials and Methods

### Study area

The study was conducted at Guangzhou Fruit World (23°20'24.72"N, 113°32'12.13"E). This research, and tourist facility and park, which was established by Guangzhou Fruit Research Institute in 1996, is located in the hilly land region of Guangdong Province, southern China. The climate of this area is subtropical oceanic monsoon with a mean temperature of 13.3 °C in January and 28.4 °C in July. The mean annual precipitation is 1694 mm and the annual average sunshine duration is 1900 hours. The soil in this area is classified as Ultisol according to USDA soil taxonomy [20].

### Experimental design

Four orchards were investigated, and these contained sweetsop (*Annona squamosa*), lichee (*Litchi chinensis*), longan (*Dimocarpus longan*), and guava (*Psidium guajava*) trees. The sweetsop orchard occupied 3.0 hectares and had 780 trees per hectare; in 2008, when soil samples were collected (see next section), The sweetsop trees were 2.5±0.1 m in height, 16.1±0.9 cm in DBH (diameter at 30 cm height above the ground), and 4.1±0.2 m in crown diameter. The lichee orchard occupied 6.2 ha and had 495 trees per ha; in 2008, the lichee trees were 5.9±0.1 m in height, 21.9±1.0 cm in DBH, and 4.7±0.3 m in tree crown diameter. The longan orchard occupied 5.4 ha and had 265 trees per ha; in 2008, the longan trees were 6.7±0.1 m in height, 31.9±1.8 cm in DBH, and 8.5±0.5 m in crown diameter. The guava orchard occupied 1.67 ha and had 809 trees per ha; in 2008, the guava trees were 1.9±0.1 m in height, 13.5±0.5 cm in DBH, and 3.5±0.2 m in crown diameter. The understory vegetation across the four orchards was quite similar and was dominated by *Axonopus compressus*, *Polygonum chinense*, *Patrinia villosa*, *Spermacoce latifolia*, *Ageratum conyzoides*, *Bidens pilosa*, and *Amaranthus viridis*. The total plant biomass of understory vegetation in 2008 was 868.7±197.9 g m^-2^ in the sweetsop orchard, 322.0±66.9 g m^-2^ in the lichee orchard, 253.1± 65.3 g m^-2^ in the longan orchard, 508.3±70.2 g m^-2^ in the guava orchard. In each of the four orchards, one-third of the total orchard area was randomly assigned to receive a clean-tillage treatment, and the understory vegetation in that area was periodically removed with a mower. The remaining two-thirds of the area of each orchard system was used as sod culture treatment. The other management practices for both treatments were uniform across the four orchards. The clean-tillage and sod culture treatments were initiated in 1998 and have been maintained since that time.

### Soil sampling and analysis

In March 2008, 10 years after we established the treatments, we designated three 15 m × 15 m plots in each treatment area for soil sampling. Soil samples were collected at five depths in each plot: 0-20, 20-40, 40-60, 60-80, and 80-100 cm. Five soil cores from random sampling points in each plot were combined to provide one composite soil sample per plot, and three composite samples per treatment area. After the surface organic materials and visible roots were carefully removed, each composite sample was passed through a 2-mm sieve and air-dried for chemical analysis. The methods used for the analysis of soil physicochemical properties were adopted from Lu [21]. The bulk density of the soils was determined by the core ring method. Soil organic carbon (SOC) was determined by the potassium dichromate titration and digestion method. Total nitrogen (TN) was determined by the semimicro Kjeldahl method. Total phosphorus (TP) was determined colorimetrically after wet digestion with H_2_SO_4_ plus HClO_4_. Total potassium (TK) was determined by the Na _2_CO_3_ extraction-flame photometer method. Soil carbon stock in top 1m soil profile was calculated as:, where the soil carbon stock is the cumulative soil carbon stock at a fixed soil depth to the bottom of n layers (t C ha^−1^), SOC (i) is the soil organic carbon concentration in the *i* th layer (g C kg^-1^), BD (i) is the bulk density in the *i* th layer (g cm^-3^), and TH (i) is the thickness of the *i* th layer (cm).

In 2008, an economic survey was also conducted of 50 fruit producers from local rural communities in the form of semi-structured interviews plus a questionnaire. Information about economic costs and benefits of fruit production was gathered during the survey. The information about economic costs included fruit seedlings, fertilization, pesticides, gasoline, machines, electric power, labor, and fruit packaging. The information about economic benefits included fruit yield and market price. The gathered economic information was converted into Chines Yuan according to the market price.

### Statistical analysis

Three-way ANOVAs were used to test the effects of understory management (sod culture vs. clean tillage), orchard type, soil depth and their interactions on SOC, TN, TP and TK. Two-way ANOVAs were used to test the effects of understory management, orchard type and their interactions on soil carbon stock (1 m depth). A paired *t*-test was used to examine the economic benefit of sod culture in subtropical orchards. All univariate analyses were performed in SPSS Statistics 20.0 (SPSS. Inc, Chicago, IL).

## Results

### Soil fertility

**Figure 2:**
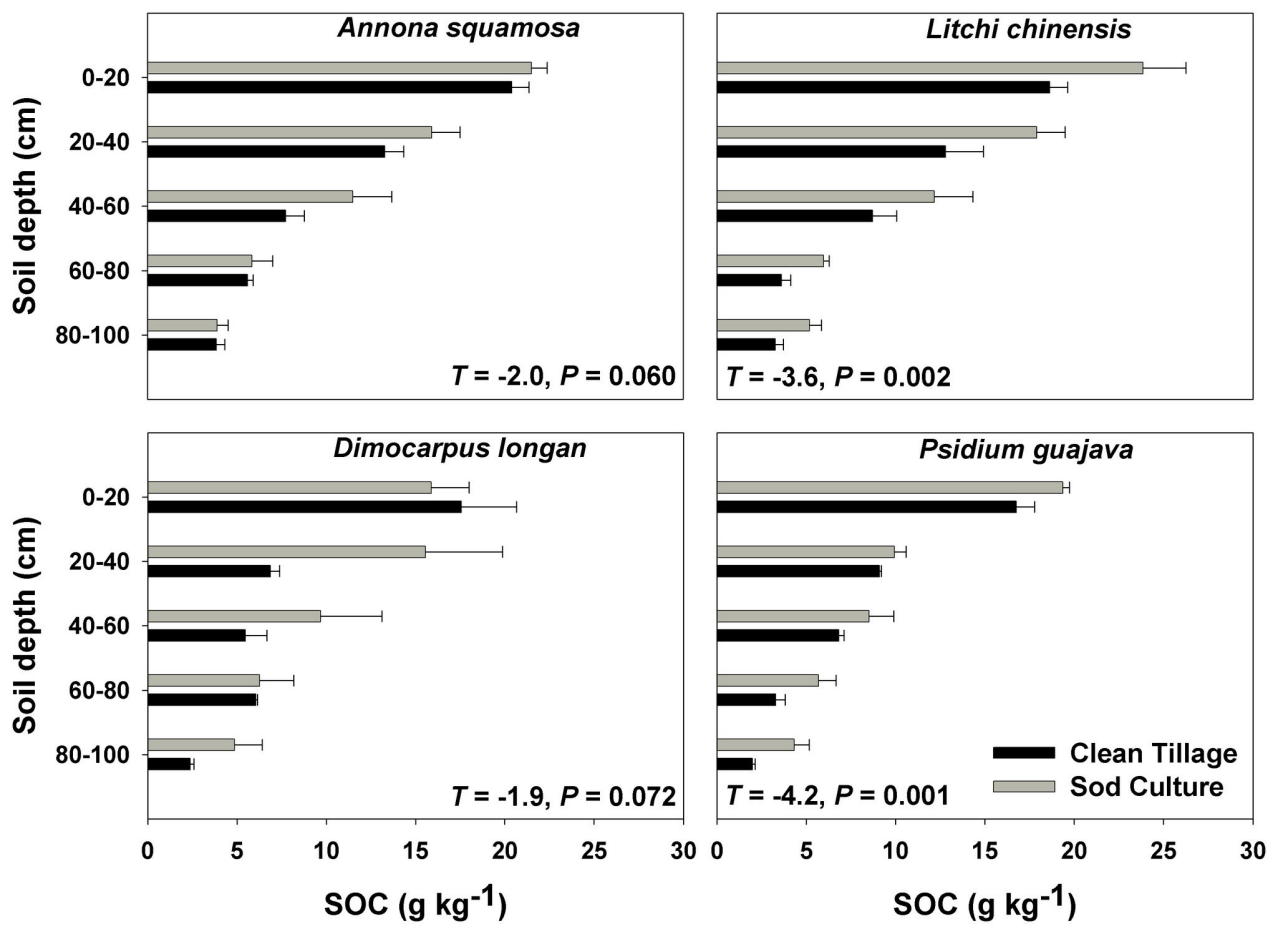
Soil organic carbon content in four subtropical orchards as affected by understory management. Values are means + SE of three replications. *T* values and *P* values are from paired *t*-tests.

**Figure 3:**
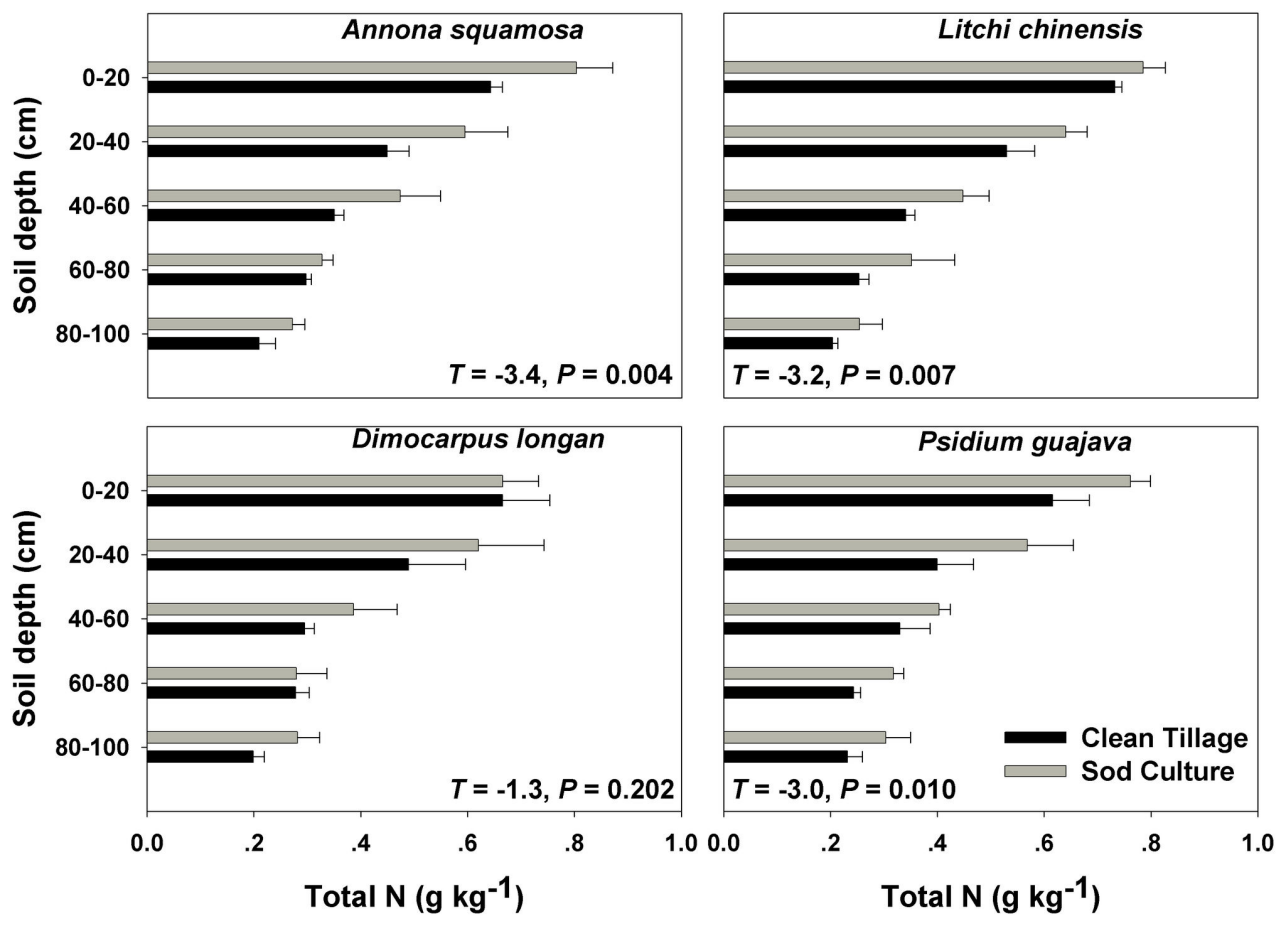
Soil total nitrogen content in four subtropical orchards as affected by understory management. Values are means + SE of three replications. *T* values and *P* values are from paired *t*-tests.

**Figure 4:**
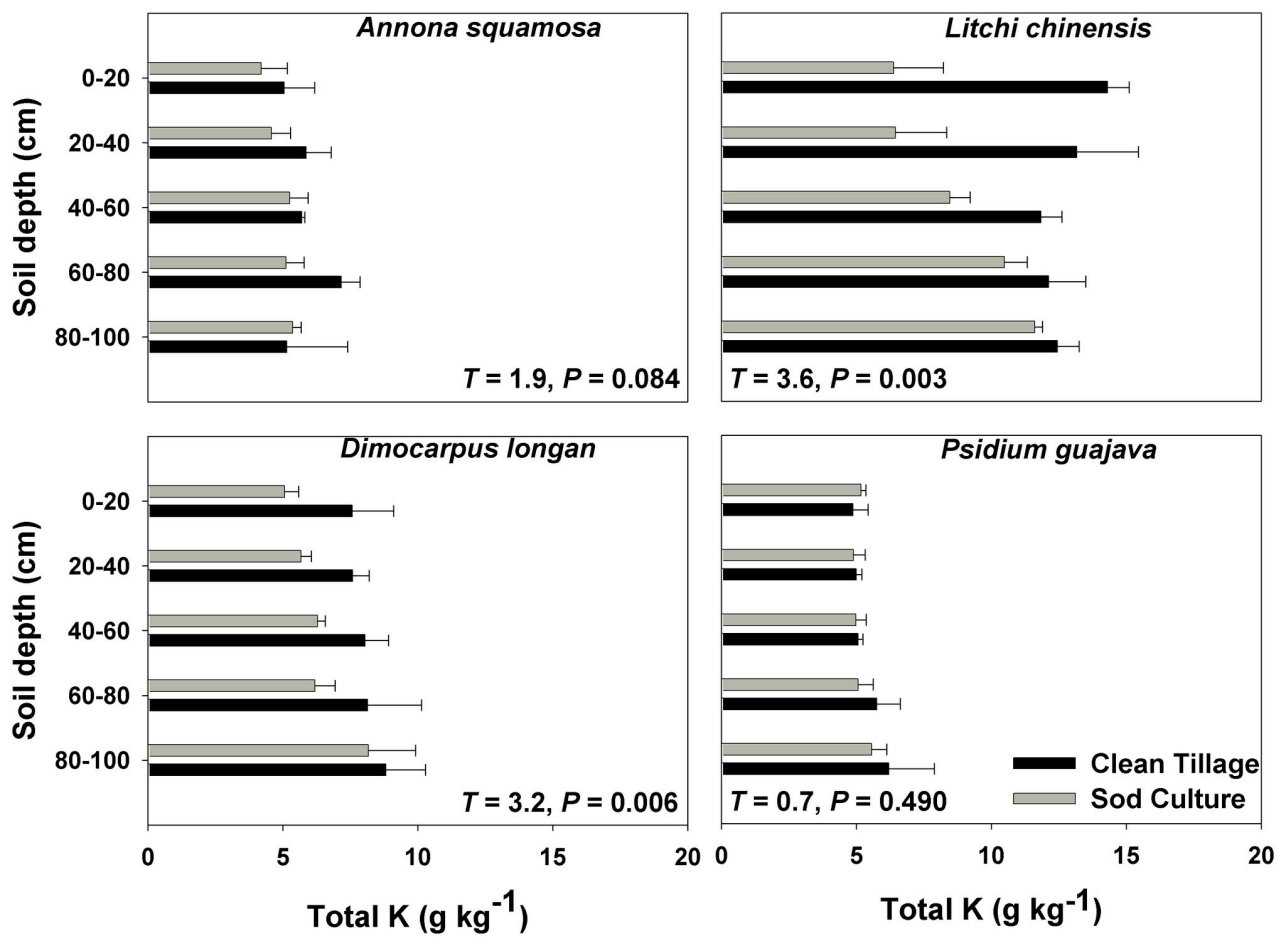
Soil total potassium content in four subtropical orchards as affected by understory management. Values are means + SE of three replications. *T* values and *P* values are from paired *t*-tests.

**Figure 5:**
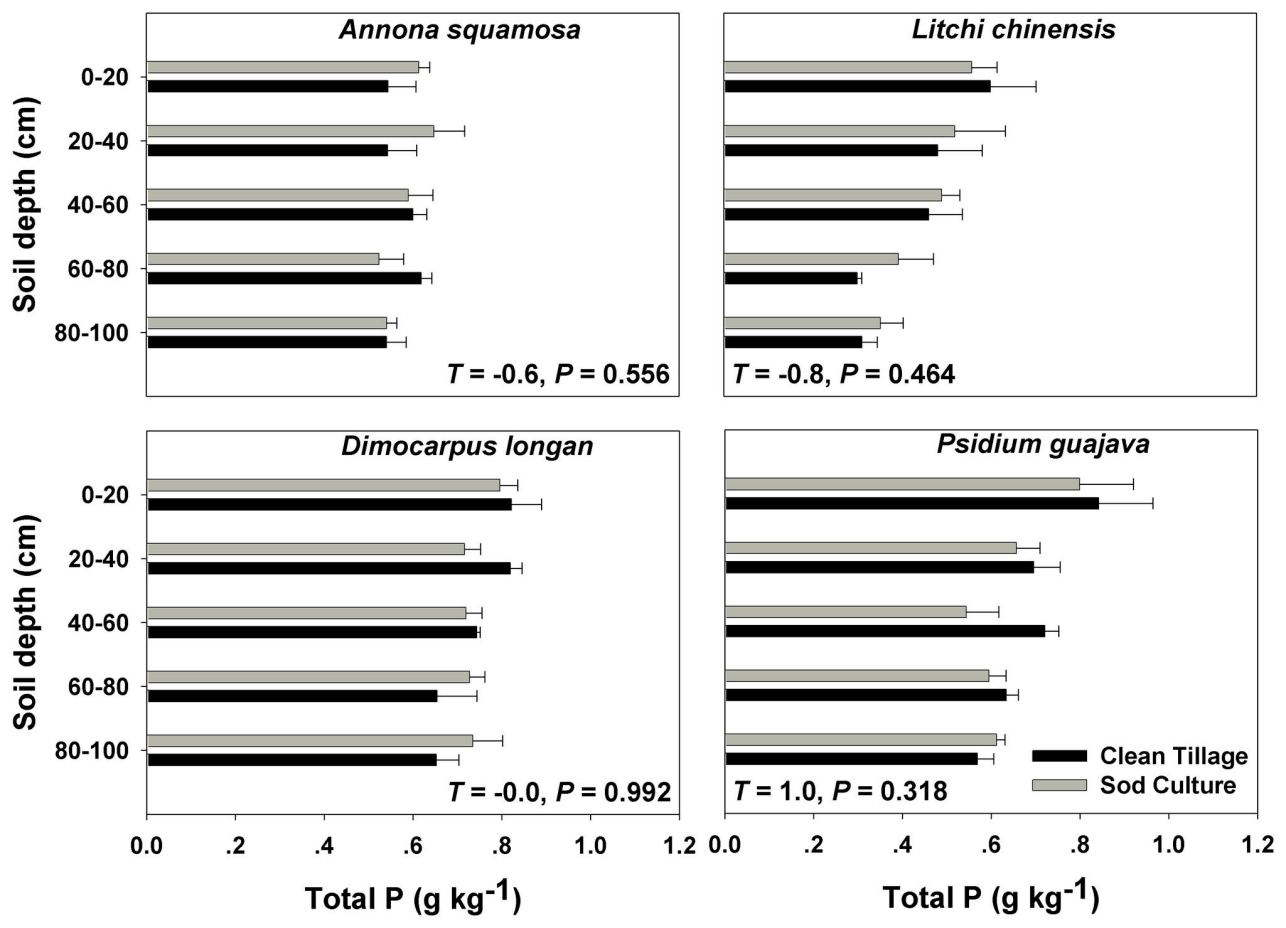
Soil total phosphorus content in four subtropical orchards as affected by understory management. Values are means + SE of three replications. *T* values and *P* values are from paired *t*-tests.

Understory management significantly influenced soil fertility in the top 1 m of the soil profile in the four orchards. SOC was greater in sod culture plots than in clean-tillage plots (Figure 2 and Table 1). Similarly, total soil N was greater in sod culture plots than in clean-tillage plots (Figure 3 and Table 1). Total soil K was greater in clean-tillage plots than in sod culture plots (Figure 4 and Table 1) while total P did not differ between the two treatments (Figure 5 and Table 1). Orchard type significantly affected SOC, total P, and total K, but not total N (Table 1). Soil sampling depth significantly affected SOC, total N, and total P (Table 1). Total K was also significantly affected by the interaction of understory management × orchard type (Table 1).

**Table 1:** Effects of understory management (sod culture vs. clean-tillage), orchard type, soil depth, and two-way interactions on soil fertility.

	Response variable					
Soil fertility variable	Understory management (UM)	Orchard type (OT)	Soil depth (SD)	UM × OT	UM × SD	OT × SD
SOC	**26.6 (<0.001)**	**7.6 (<0.001)**	**135.1 (<0.001)**	0.9 (0.450)	1.4 (0.243)	1.4 (0.167)
TN	**27.6 (<0.001)**	1.2 (0.315)	**101.1 (<0.001)**	0.4 (0.760)	0.8 (0.542)	0.6 (0.853)
TP	0.0 (0.942)	**42.4 (<0.001)**	**8.7 (<0.001)**	0.9 (0.473)	0.5 (0.710)	1.2 (0.325)
TK	**26.6 (<0.001)**	**57.0 (<0.001)**	2.3 (0.065)	**6.2 (<0.001)**	1.5 (0.224)	0.4 (0.966)

*F* values (and *P* values) are from univariate ANOVAs. Statistically significant values (*P* < 0.05) are shown in bold. Degrees of freedom for *F* values are 1, 80 for understory management; 3, 80 for orchard type and UM × OT; 4, 80 for soil depth and UM × SD; and 12, 80 for OT × SD.

### Soil carbon sequestration

**Figure 6:**
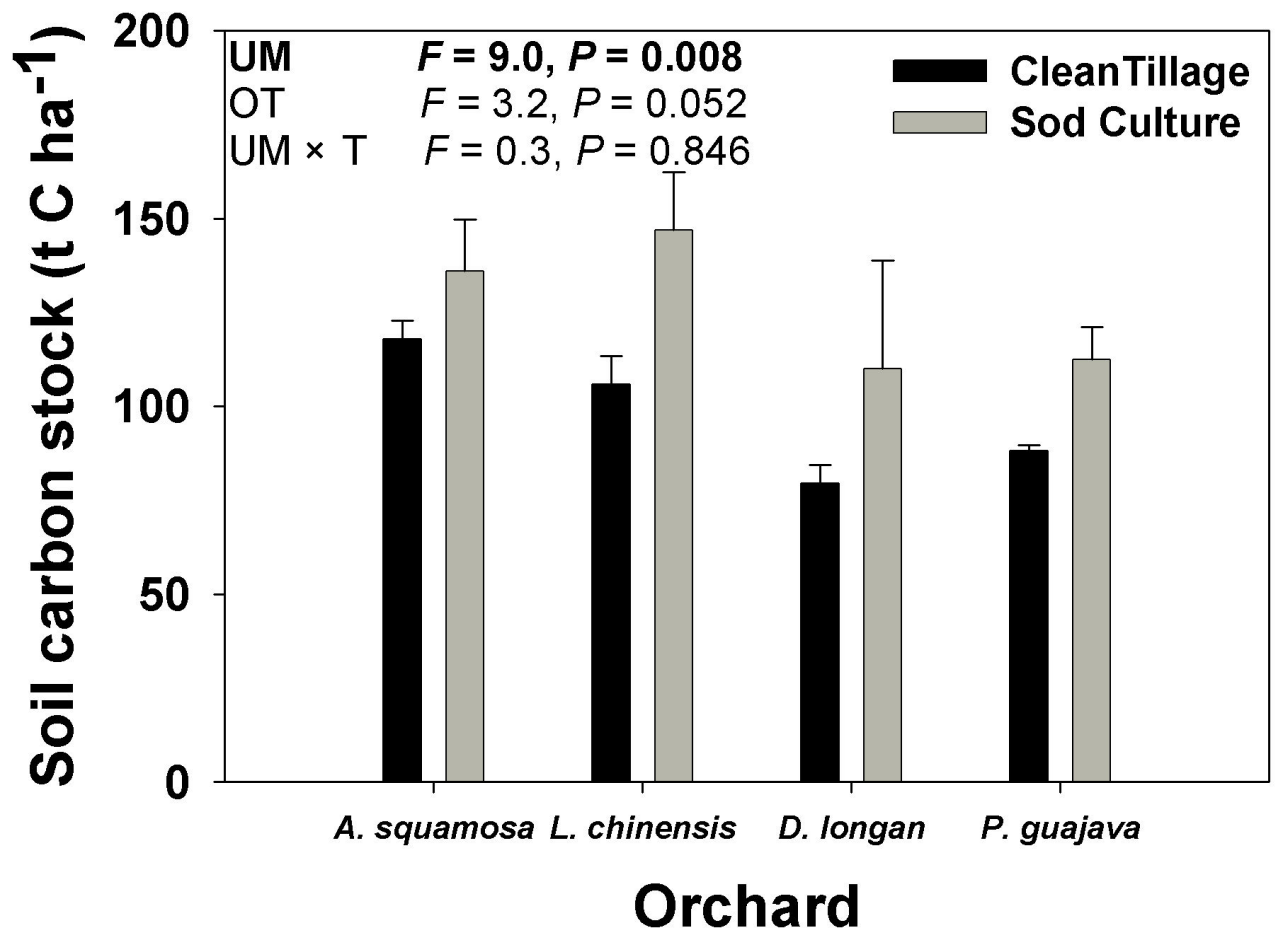
Soil carbon stock in four subtropical orchards as affected by understory management (UM), orchard type (OT), and their interaction. Values are means + SE of three replications. *F* values and *P* values are from two-way ANOVAs. *P* values <0.05 are shown in bold.

The C stock in the top 1 m of soil was significantly greater with sod culture than with clean tillage (Figure 6). Soil C stock, however, was not affected by orchard type or the interaction of orchard type × understory management. Given that the treatments had been differentiated 10 years earlier, sod culture increased annual C sequestration in the upper 1 m of soil by 2.85 t C ha^-1^ year^-1^ relative to clean tillage

### Economic benefit analysis

**Figure 7:**
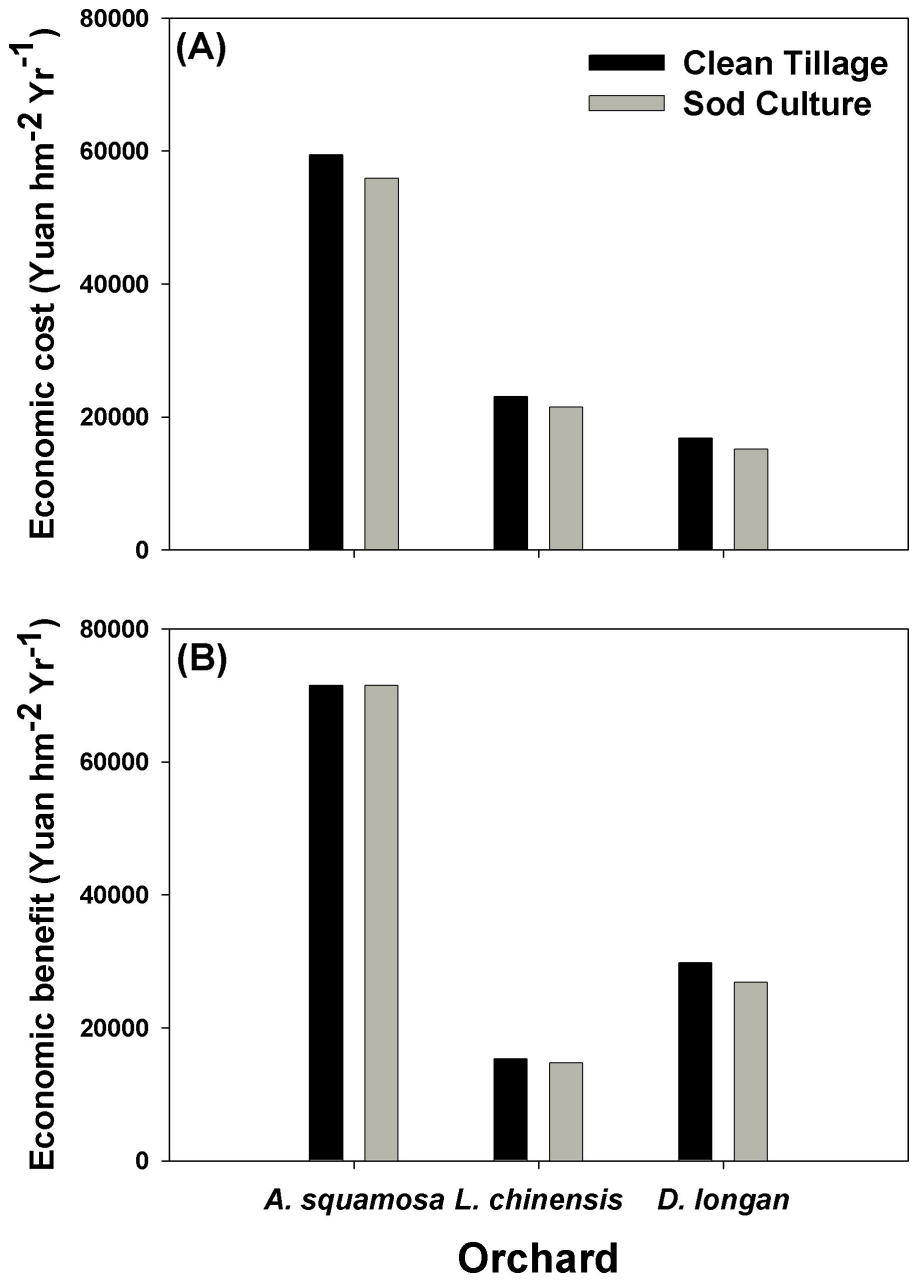
Effects of understory management on the economic costs (A) and benefits (B) in three subtropical orchards.

Sod culture decreased economic costs by 6-10% relative to clean tillage but the decrease was not statistically significant (Figure 7A: *T* =3.4, *P* = 0.075). The economic benefit was slightly less with sod culture than with clean tillage but the difference was not statistically significant (Figure 7B: *T* = 1.3, *P* = 0.315).

## Discussion

Our study has several implications. First, it provides experimental evidence that the introduction of sod culture to subtropical orchards can increase soil fertility, which is beneficial for the sustainability of orchard production. Second, it demonstrates that maintaining a living understory covered by sod culture in subtropical orchards can greatly enhance soil C storage and shows a promising potential for increasing soil carbon sequestration compared with clean tillage. Third, our study also highlights that introducing sod culture offers great promise to sequestrate more C in soils considering that China has the largest area of orchards in the world and few of these orchards currently have sod understories.

Understory vegetation can act as a potential source of soil nutrients and has been implemented as the floor management practice in orchard systems [22]. Our results showed that maintaining a living understory cover by sod culture greatly enhanced soil organic carbon and total nitrogen content in four subtropical orchard systems over a ten-year experiment. Previous studies about orchard floor management also found that sod culture increased soil fertility [13]. The enhancement of soil fertility by sod culture may be explained as follows: (1) maintenance of living understory cover by sod culture can produce large amount of plant residues which contribute to the formation of humus and the accumulation of soil organic matter [23]; (2) Sod culture can greatly reduce soil nutrient loss by reducing surface runoff [16,24,25]; and (3) Sod culture can reduce the frequent mechanical disturbance of soils and thereby reduce soil organic matter decomposition [26,27]. In addition, our data showed that although sod culture increased the total soil N content, it significantly reduced the total K content in the upper 1 m soil profiles of the soils, suggesting that there is a strong competition for K between overstory trees and understory cover [28]. It also highlights the importance of K fertilizers from the management perspective. Therefore, reasonable K fertilizer application should be taken into account if sod culture is maintained in similar soils of South China.

Maintaining a living understory cover by sod culture showed promising soil C sequestration in subtropical orchards. Averaged across the four orchards in the current study, the C stock in the top 1 m of soil was 28.5 t ha^-1^ greater in the sod culture plots than in the clean-tillage plots. Assuming that C stocks were similar in the plots at the start of the experiment and given that the treatments had been maintained for 10 years, we estimate that sod culture increased C sequestration in the top 1 m of soil by 2.85 t ha^-1^ y^-1^. Furthermore, the ecological benefits in term of soil carbon sequestration did not significantly affect the economic performance of orchard production. C inputs to soil are determined by the amount and distribution of primary production, the life cycle of the vegetation, and exogenous organic matter additions. Thus, practices that increase net primary production and/or return a greater portion of plant materials to the soil have the potential to increase soil C stocks. Practices that reduce the decomposition rate by altering these physical, chemical, or biological controls also lead to carbon storage.

It has been well acknowledged that maintenance of a living understory cover by sod culture can increase C inputs from understory plant residues, prevent soil erosion and reduce soil disturbance [24], which has shown to increase soil C storage and reduce the decomposition rate of soil organic matter. Given that the extent of orchard plantings in China and given that few of these orchards maintain a sod culture, the promising potential of soil C sequestration through sod culture is very important for management application of orchard systems in terms of soil C sequestration. In Guangdong Province, for example, the orchard area in 2009 was 1.096 × 10^6^ ha; if sod culture were applied in all the orchards of this province, soil C sequestration could theoretically be increased by 3.12 Tg C y^-1^.

The orchard understory represents an important component of the orchard systems, but it has generally received less attention on soil C sequestration than have tree horticulture and pest management [29]. In the face of global climate change, fruit producers must adapt their management practices to deal with the changing conditions. Fruit producers also need C sequestration techniques that provide multiple benefits while reducing atmospheric CO_2_ concentration at lower overall cost. A plant-based solution to orchard understory management would be ideal from a sustainability standpoint. Management of agricultural systems to sequester C has been accepted as a partial solution to climate change [30]. Our research clearly indicates that maintaining a living understory cover by sod culture has great potential to enhance soil C sequestration while providing numerous environmental, economic and social benefits, and should be considered as a management option to meet the greenhouse gas emission reduction goal in subtropical orchard systems. The implementation of sod culture as an option for C mitigation in orchard systems could be justified for many other reasons. First, increased soil C sequestration greatly benefits the sustainability of orchard production by maintaining soil fertility. Second, given the improbability of obtaining any single mitigating method, maintaining a living understory cover by sod culture to enhance soil C sequestration appears to be a more realistic way of achieving CO_2_ reduction targets. Third, the financial cost of C sequestration through sod culture appears to be much lower compared to most other CO_2_ mitigating options. The costs of sod culture could be easily offset by the monetary benefits from orchard products and trading in C credits. Finally, sod culture is a “clean” and social-friendly technique and has minimal environmental and health risks.

Although sod culture increases soil C sequestration, increases soil fertility, and reduces soil erosion, sod culture does not increase orchard production costs based on our analysis (Figure 7A). This finding should encourage growers in southern China to introduce and maintain sod cultures in their orchards. It should be pointed out, however, that soil C sequestration is a result not only of management, but also of soil and climatic conditions [31]. The effect of maintaining a living understory cover by sod culture on soil C sequestration should be tested over a wide range of soil types, climates, and fruit tree species. Making decisions about the feasibility of sod culture also requires an interdisciplinary approach to exploring the biophysical, technical, economic, and practical potential of management options to soil C sequestration.
